# Effect of Antioxidants Supplementation on Aging and Longevity

**DOI:** 10.1155/2014/404680

**Published:** 2014-03-25

**Authors:** Izabela Sadowska-Bartosz, Grzegorz Bartosz

**Affiliations:** ^1^Department of Biochemistry and Cell Biology, University of Rzeszów, Zelwerowicza Street 4, 35-601 Rzeszów, Poland; ^2^Department of Molecular Biophysics, University of Łódź, Pomorska 141/143, 90-236 Łódź, Poland

## Abstract

If aging is due to or contributed by free radical reactions, as postulated by the free radical theory of aging, lifespan of organisms should be extended by administration of exogenous antioxidants. This paper reviews data on model organisms concerning the effects of exogenous antioxidants (antioxidant vitamins, lipoic acid, coenzyme Q, melatonin, resveratrol, curcumin, other polyphenols, and synthetic antioxidants including antioxidant nanoparticles) on the lifespan of model organisms. Mechanisms of effects of antioxidants, often due to indirect antioxidant action or to action not related to the antioxidant properties of the compounds administered, are discussed. The legitimacy of antioxidant supplementation in human is considered.

## 1. Introduction

Aging is an unavoidable, universal, biological phenomenon affecting all multicellular organisms (with few apparent exceptions) and probably common also among unicellular organisms, including protozoa, yeast, and bacteria [1, 2]. Although different hypotheses have been put forward to explain the cellular and molecular mechanisms of aging, recent studies made it increasingly clear that aging is due to accumulation of molecular damage, giving rise to a unified theory of aging [3–8]. Among reactions contributing to this damage, reactions of free radicals and other reactive oxygen species are the main reason, apart from reactions of metabolites such as sugars and reactive aldehydes and spontaneous errors in biochemical processes [9].

From a thermodynamic point of view, all aerobic organisms are subject to the action of common oxidant, that is, oxygen. The redox potential of the O_2_/2H_2_O redox system (approximately + 0.8 V at pH 7) is more positive than those of most other biologically relevant redox systems. Therefore, the oxidation by O_2_ of organic compounds will have a negative free enthalpy and should proceed spontaneously. In other words, organic compounds and structures composed of them are thermodynamically unstable in an oxygen-containing atmosphere. Molecular oxygen, in its triplet basal state, is rather unreactive due to the spin restriction. However, formation of oxygen free radicals and other reactive oxygen species (ROS) opens the gate for potentially deleterious oxidative reactions of oxygen [7]. Seen from that perspective, the “Free Radical Theory of Aging” (FRTA) [10], now more commonly termed the oxidative damage theory of ageing, seems to address a key facet of intrinsic biological instability of living systems [11, 12]. The basic idea of the FRTA is that free radicals and other ROS, formed unavoidably in the course of metabolism and arising due to the action of various exogenous factors, damage biomolecules, and accumulation of this damage are the cause of age-related diseases and aging.

If FRTA is true, antioxidants should slow down aging and prolong lifespan. This apparently obvious conclusion has stimulated enormous number of studies aimed at finding a relationship between levels of endogenous antioxidants and lifespan of various organisms on the effects of addition of exogenous antioxidants on the course of aging and lifespan of model organisms. Pubmed provides more than 13300 hits for conjunction of terms “antioxidant” and “aging or ageing.” However, in spite of the plethora of studies, the answer to the question if exogenous antioxidants can prolong life is far from being clear.

## 2. Effect of AOs on the Lifespan of Model Organism

**Figure 1:**
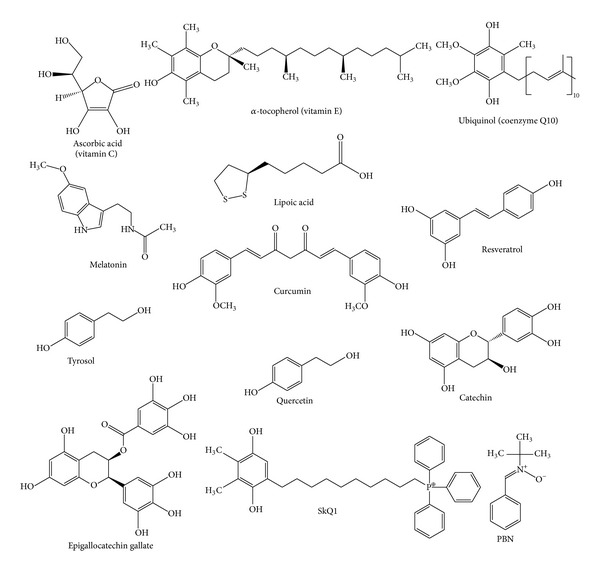
Some antioxidants studied as antiaging agents.

Many studies have addressed the question of supplementation with antioxidant vitamins, especially vitamins C and E, and synthetic compounds can prolong the lifespan of model animals. Vitamin C (ascorbic acid) is the major hydrophilic antioxidant and a powerful inhibitor of lipid peroxidation. In membranes, this molecule rapidly reduces *α*-tocopheroxyl radicals and LDL to regenerate *α*-tocopherol and inhibit propagation of free radicals. Vitamin E (*α*-tocopherol) is the main hydrophobic antioxidant in cell membranes and circulating lipoproteins. Its antioxidant function is strongly supported by regeneration promoted by vitamin C. Vitamin E is thought to prevent atherosclerosis through inhibition of oxidative modification. Coenzyme Q (ubiquinol, CoQ) and lipoic acid in their reduced forms and melatonin (Figure 1) are also efficient antioxidants.

Novel endogenous indole, indolepropionamide, another endogenous antioxidant, is similar in structure to melatonin, binds to the rate-limiting component of oxidative phosphorylation in complex I of the respiratory chain, and acts as a stabilizer of energy metabolism, thereby reducing ROS production [13].

Epitalon is a synthetic tetrapeptide Ala-Glu-Asp-Gly, showing antioxidant activity [14]. (S,S)-6-hydroxy-2,5,7,8-tetramethylchroman-2-carbonyl-beta-alanyl-L-histidine (*S,S*-Trolox-carnosine) is a synthetic analogue of carnosine containing a Trolox (water-soluble analog of vitamin E) residue [15].

Recently, the antiaging effect of resveratrol (RSV) has been a hotly discussed topic. RSV was first isolated from the roots of white hellebore (*Veratrum grandiflorum, O. Loes*) in 1940 and later in 1963 from the roots of* Polygonum cuspidatum *(or* Fallopia japonica*), a plant used in traditional Chinese and Japanese medicine [16]. This polyphenolic compounds are a phytoalexin that stimulates cell defenses in plants. RSV is synthesized in many plants, such as peanuts, blueberries, pine nuts, and grapes, which protects them against fungal infection and ultraviolet irradiation. It mainly accumulates in a glycosylated state (piceid). Some dimethoxylated RSV derivatives (pterostilbene) are also present as well as RSV oligomers (**ε**-viniferin, a dimer, and hopeaphenol, a tetramer). Interestingly, RSV plays a number of protective roles in animals, although it is rapidly metabolized in a conjugated form (glucorono- or sulfo-) [17]. Since the early 1990s, it has been suggested that RSV could be the molecule responsible for the French paradox, that is, the low occurrence of coronary heart diseases and cardiovascular diseases in South-Western France, despite the consumption of a high saturated fat diet. The French paradox was correlated to some extent with the regular consumption of red wine, which contains high levels of RSV [18].

Curcumin [1,7-bis (4-hydroxy-3-methoxyphenyl)-1,6-heptadiene-3,5-dione] (diferuloylmethane, CUR), the main component of the yellow extract from the plant* Curcuma longa* (turmeric, a popular Indian spice), is a main bioactive polyphenol, which has been used widely as a spice, food additive, and a herbal medicine in Asia [19]. Tetrahydrocurcumin (THC) is an active metabolite of CUR. Orally ingested CUR is metabolized into THC by a reductase found in the intestinal epithelium. THC possesses extremely strong antioxidant activity compared to other curcuminoids. The antioxidant role of THC has been implicated in recovery from renal injury in mice and in anti-inflammatory responses [20]. Tyrosol is a main phenol present in extra virgin olive oil [21].

Some researchers hope that development of new means of introduction of antioxidants into cells or construction of new antioxidants can make a breakthrough in antioxidant modulation of aging and longevity. If mitochondria are the main source of ROS in the cell, mitochondrially targeted antioxidants could be more effective than traditional ones. This idea was the basis of synthesis of positively charged derivatives of plastoquinone and other antioxidants which are retained in the mitochondria due to the high negative potential at the inner mitochondrial membrane [22]. SkQ1 is a mitochondria-targeted, plastoquinone-containing [10-(6′-plastoquinonyl) decyltriphenylphosphonium] [23].

Results of studies on the supplementation of model organisms with antioxidant vitamins and other antioxidants are divergent. Examples of recent studies devoted to this question are summarized in Table 1 and these data are only commented in this section.

Ascorbic acid partially rescued the lifespan of superoxide dismutase (SOD)-deficient yeast* Saccharomyces cerevisiae *which was considerably reduced as a result of lack of this vital antioxidant enzyme [12]. However, this effect should be seen rather as a partial restoration of the redox status seriously deranged in these cells rat compared to life extension of normal yeast cells. Another study, using but D-erythroascorbic acid (ascorbic acid homologue produced in the yeast) showed little effect of this antioxidant on the replicative lifespan of wild-type yeast [13]. Similar reports have been published for multicellular organisms, in which antioxidants had life-prolonging effects on mutants deficient in antioxidant defense or were subjected to oxidative stress but did not affect the lifespan of healthy wild type animals.

Supplementation of the growth medium of* S. cerevisiae* with the lipophilic antioxidants *α*-tocopherol and CoQ alone, or in combination with *α*-tocopherol, increased oxidative stress and decreased cellular lifespan [24]. It should be recalled, however, that* S. cerevisiae* is unable to produce polyunsaturated fatty acids [25] so lipid oxidative damage may be of lower importance and lack of protective effects of hydrophobic antioxidants, located mainly in cell membranes [24], maybe not surprising in this species.

Effect of vitamin C on the lifespan of several multicellular model organisms (*Caenorhabditis elegans, Drosophila melanogaster,* mice, rats, and guinea pigs) has been recently reviewed by Pallauf et al. No consistent picture emerges from the summary of data, some studies demonstrating prolongation of lifespans and others showing no effect [26]. Ernst et al. conducted a comprehensive literature review regarding the effect of vitamin E on lifespan in model organisms including single-cell organisms, rotifers,* C. elegans*,* D. melanogaster,* and laboratory rodents. The findings of their review suggest that there is no consistent beneficial effect of vitamin E on lifespan in model organisms, which corresponds to results of meta-analysis of mortality in human intervention studies [27].

While most of the studies concerning mammals have been done on mice, an interesting study has addressed the effect of dietary supplementation with either vitamin E or vitamin C (ascorbic acid) on a wild-derived animal, short-tailed field vole (*Microtus agrestis*). Antioxidant supplementation for nine months reduced hepatic lipid peroxidation, but DNA oxidative damage to hepatocytes and lymphocytes was unaffected. Surprisingly, antioxidant supplementation significantly shortened lifespan in voles maintained under both cold (7 ± 2°C) and warm (22 ± 2°C) conditions [28].

Hector et al. (2012) quantified the current knowledge of life extension of model organisms by RSV. These authors used meta-analysis techniques to assess the effect of RSV on survival, using data from 19 published papers, including six species: yeast, nematodes, mice, fruit flies, Mexican fruit flies, and turquoise killifish. While the lifespan of the turquoise killifish was positively affected by the RSV treatment, results are less clear for flies and nematodes, as there was important variability between the studies [29].

The rapid expansion of nanotechnology provided a huge assortment of nanoparticles (NPs) that differ in chemical composition, size, shape, surface charge and chemistry, and coating and dispersion status. Antioxidant delivery can be significantly improved using various NPs [30]; some NPs possess antioxidant properties and are able to efficiently attenuate oxidative stress by penetrating specific tissues or organs, even when administered at low concentrations and found to increase the lifespan of model organisms [31, 32]. Nevertheless, there is an increasing concern about the toxicity, especially genotoxicity of NPs, and this question field requires thorough studies.

It has been argued that antioxidant mixtures, such as those found in natural products, are better than simple antioxidant formulas, that is, due to synergism between antioxidants. KPG-7 is a commercially available herb mixture containing* Thymus vulgaris*,* Rosmarinus officinalis*,* Curcuma longa*,* Foeniculum vulgare*,* Vitis vinifera* (polyphenol), silk protein,* Taraxacum officinale,* and* Eleutherococcus senticosus*, which have been reported to include a variety of antioxidant, antitumoral, and anti-inflammatory bioactivities. Positive effects of such extracts on the lifespan of model organisms have been reported but other studies showed no significant effects. For example, administration of a complex mixture of vitamins, minerals, botanical extracts, and other nutraceuticals, rich in antioxidants and anti-inflammatories, to male mice starting from the age of 12 m, failed to affect their lifespan [33].

In the honeybee* Apis mellifera* L., queens live and reproduce for 1–4 years but hive workers, which are derived from the same diploid genome, live for only 3–6 weeks during the spring and summer. Queens are fed throughout their lives with royal jelly, produced by the hypopharyngeal, postcerebral, and mandibular glands of the worker bees. In contrast, workers are fed royal jelly for only a short period of time during their larval stages. It suggests that royal contains longevity-promoting agents for queens which may perhaps affect the longevity of other species if it affects the “public” mechanisms of aging [34]. However, the effect of royal jelly on the maximal lifespan of mice was rather disappointing [35]. Moreover, the action of complex preparations including plant extracts is difficult to interpret because, apart from antioxidants, they contain various biologically active products [36].

## 3. Reversal of Age-Related Changes by Antioxidants

Apart from the effect of prolongation of lifespan by antioxidant administration throughout most of the lifetime (long-lasting experiments), another approach to study antiaging effect of antioxidants consists in short-time experiments, in which functional tests compare the status of experimental animals before and after supplementation. An experiment of this type consisted in administration of N-*tert*-butyl-*α*-phenylnitrone (PBN) to aged Mongolian gerbils for 2 weeks. Such a treatment reduced the amount of protein carbonyls in brain, augmented the activity of glutamine synthetase, and decreased the number of errors in radial arm maze patrolling behavior, normalizing the values to those typical for young animals. However, these changes were reversible after cessation of PBN treatment [37]. Similarly, relatively old mice (17.5 months) fed high-CoQ diet (2.81 mg/g) for 15 weeks improved special performance in Morris water maze test and reduced protein oxidative damage [38].

## 4. How Do “AOs” (Do Not) Act? Possible Explanations

Generally, the effects of antioxidant supplementation in model organisms are disappointing. Many studies showed no effect or even negative effects on the lifespan. Only in some cases considerable prolongation of lifespan was obtained and in organisms which are evolutionarily quite distant from mammals. In some cases, mean but not maximal lifespan was affected, which may be caused by reduction of mortality due to diseases rather than interference with the aging process itself. An apparently obvious conclusion from the plethora of studies could be that antioxidants cannot be expected to prolong significantly the lifespan, especially of mammals, which does not support the FRTA.

However, perhaps such a simple conclusion would be precocious, not taking into account experimental setup employed in different studies. One of the questions is the relevance of use of model organisms if understanding human aging is aimed. The basic biochemical mechanisms are so common in all living world that there are good reasons to expect that the mechanisms governing aging are also universal. It may not always be true. It has been suggested that there are “public” and “private” mechanisms of aging [39]. Seemingly, the mechanisms of aging of* S. cerevisiae*, used as a model organism in biogerontology, may be rather private than public. This refers to both “chronologic” aging where yeast survival is limited by exhaustion of nutrients and/or accumulation of toxic products of metabolism and to “replicative” aging which seems to be a measure of fecundity rather than longevity and is limited by other factors compared to those relevant to aging of multicellular organisms [40, 41]. Somatic cells of* C. elegans* and* D. melanogaster* are postmitotic, which only partly reflects the situation in mammalian tissues.

It should be taken into account that ascorbic acid, which is a vitamin for primates, is synthesized by other organisms including mice and rats [42]. It does not preclude the antioxidant action of ascorbate in these organisms but administration of exogenous ascorbic acid may inhibit its endogenous synthesis.

Sometimes the administered antioxidants may be not fully taken up especially when added to complex media. Numerous studies using* C. elegans* have used a protocol, in which chemicals are orally delivered by incorporating them into the nematode growth media or mixing with the food bacteria. However, actual exposure levels are difficult to estimate. The use of liposomes loaded with water-soluble substances resulted in successful oral delivery of chemicals into the intestines of* C. elegans*. When using liposomes, oral administration of hydrophilic antioxidants (ascorbic acid, N-acetyl-cysteine, reduced glutathione, and thioproline) prolonged the lifespan of the nematodes, whereas the conventional method of delivery showed no longevity effects [43]. It is also difficult to estimate the amount of ingested food in many model organisms, such as* C. elegans* or* D. melanogaster*, so the effects of admixture of high doses of antioxidants may lead feeding rejection and thus starvation [44].

The life-prolonging effect of antioxidants may be limited to a more or less narrow “therapeutic window”. This window may be different for various organisms, that is, due to differences in the uptake rate and metabolism. Not always, the experimental conditions may hit the therapeutic window.

Introduction of antioxidants in the diet may affect the endogenous antioxidant system and the effect is not always advantageous. Farr et al. reported that supplementation with lipoic acid reduced indices of oxidative stress increasing glutathione level and decreasing the concentration of lipid peroxidation products and glutathione peroxidase activity. However, this treatment actually decreased the lifespan of SAMP8 mice [45].

The life-prolonging effect can be correlated with antioxidant properties of an additive in some but not in other cases. For example, onion flavonoids, quercetin, quercetin 3′-O-*β*-D-glucopyranoside, and quercetin 3-O-*β*-D-glucopyranoside-(4→1)-*β*-D-glucopyranoside increased the lifespan of* C. elegans* but no direct correlation was found between antioxidative activity and antiaging activity [46]. Similarly, no correlation was found between the antioxidant activities of 6 plant extracts and their lifespan benefits in* C. elegans* [47].

It should be remembered that (i) the effects of an antioxidant may be not due to its direct antioxidant action but to its indirect antioxidant effects (induction of endogenous antioxidant mechanisms) and (ii) compound called “antioxidant” may have a plethora of other effects* in vivo*, not related at all to its antioxidant action.

Antioxidants can have deleterious effects on traits that, as a consequence, increase longevity. For instance, thioproline was reported to increase longevity of* D. melanogaster* which might be ascribed to its direct antioxidant action; however, it decreased also the metabolic rate, mean weight at eclosion, and development speed of the fruit flies which might be more relevant for its life-prolonging effect [44].

Similarly, RSV reduced acute oxidative damage; however, it did not extend the normal life span of* C. elegans *indicating that antioxidant properties of this compound were probably not adequate to affect ageing [48]. Howitz and colleagues proposed that RSV is capable of increasing the deacetylase activity of human sirtuin 1 (SIRT1) [49]. SIRT1, the closest homolog of the yeast silent information regulator (sir)2 protein, functions as an NAD^+^-dependent histone and nonhistone protein deacetylase in several cellular processes, like energy metabolism, stress responses, and so forth. It has been found that RSV activates SIRT1 by increasing its binding with lamin A, thus aiding in the nuclear matrix localization of SIRT1. Ghosh et al. suggested that rescue of adult stem cell decline in laminopathy-based premature aging mice by RSV is SIRT1-dependent [50]. Besides SIRT1 activation, RSV inhibits SIRT3, and it can mimic calorie restriction/dietary restriction (DR) effects [51]. DR with adequate nutrition is the only nongenetic and the most consistent nonpharmacological intervention that extends lifespan in model organisms from yeast to mammals and protects against the deterioration of biological functions, delaying or reducing the risk of many age-related diseases. It has already been known since the 1930s that a severe lowering of calorie intake dramatically slows the rate of ageing in mammals and lowers the onset of numerous age-related diseases, including cancer, cardiovascular disease, diabetes and neurodegeneration. It is found that DR induced an 80% increase in the lifespan of unicellular organisms and some invertebrates and a 20–40% increase in small mammals [52]. The biological mechanisms of DR's beneficial effects include modifications in energy metabolism, redox status, insulin sensitivity, inflammation, autophagy, neuroendocrine function, and induction of hormesis/xenohormesis response. The molecular signalling pathways mediating the antiaging effect of DR include not only sirtuins, but also AMP-activated protein kinase (AMPK), insulin/insulin growth factor-1, and target of rapamycin (TOR/mTOR), which form a complex interacting network. Rascón et al. reported that the lifespan extension effects of RSV are conserved in the honeybee and may be driven by a mechanism related to DR. In contrast, hyperoxic stress abolished the RSV life-extension response [53].

Although RSV has been found to extend the lifespan of many model organisms including yeast, nematodes, and fruit flies in the Sir2 or (Sirtuin 2)-dependent manner, some other groups have questioned the importance of the Sir2 pathway for ageing and could not confirm a beneficial effect of RSV on the lifespan of* D. melanogaster. *A* Drosophila* strain with ubiquitous overexpression of dSir2 using the UAS-GAL4 system was long-lived relative to wild-type controls but was neither long-lived relative to the appropriate transgenic controls nor a new line with stronger overexpression of dSir2. These findings underscore the importance of controlling for genetic background and for the mutagenic effects of transgene insertions in studies of genetic effects on lifespan [48]. Burnett et al. found that DR increased fly lifespan independently of dSir2 but these findings do not necessarily rule out a role for sirtuins in determination of metazoan lifespan [54].

Marchal et al. reviewed the beneficial effects of RSV in different mammalian species, including humans, and concluded that they generally reflect the effects observed during chronic DR without malnutrition. Although most of these effects have been observed in individuals without age-associated pathology, in those, which were overweight or obese, they indicate the role of RSV in metabolic regulation and the antiaging efficacy of this intervention. One explanation is the positive and rapid changes induced by RSV, which lead to adaptive metabolic response associated with an energy balance regulation and maintenance of overall health. Moreover, data on the effects of this molecule on longevity in healthy but nonobese mammals are rare, and these authors recommend that longitudinal studies on experimental models close to humans, such as nonhuman primates, multiply [18].

Recent studies have indicated that at equivalent and diet-achievable doses pterostilbene is a more potent modulator of cognition and cellular stress than RSV, likely driven by increased peroxisome proliferator-activated receptor alpha expression and increased lipophilicity due to substitution of hydroxy with methoxy group in pterostilbene [55]. Wen et al. investigated polydatin and its role in extending lifespan, improving oxidative stress resistance and the possible regulation mechanism involved in the insulin/IGF-1 signaling (IIS) pathway. Polydatin protected against oxidative stress. It improved the expression of the inducible oxidative stress protein (GST-4) and corresponding stroke frequencies in the transgenic CL2166 strain but not due to its direct antioxidant action by mainly increased SOD-3::GFP expression in CF1553 worms and translocation of DAF-16 to the nucleus in worm cells [56].

Similarly, although CUR is a directly acting antioxidant, its lifespan-prolonging effects seem to be dependent mainly on its indirect antioxidant action (induction of antioxidant proteins) or interference with cellular signaling. CUR regulates the expression of inflammatory cytokines (e.g., TNF, IL-1), growth factors (e.g., VEGF, EGF, and FGF), growth factor receptors (e.g., EGFR, HER-2, and AR), enzymes (e.g., COX-2, LOX, MMP9, MAPK, mTOR, and Akt), adhesion molecules (e.g., ELAM-1, ICAM-1, and VCAM-1), apoptosis related proteins (e.g., Bcl-2, caspases, DR, and Fas), and cell cycle proteins (e.g., cyclin D1). CUR modulates the activity of several transcription factors (e.g., NF-*κ*B, AP-1, and STAT) and their signaling pathways [57]. Recent studies performed in both invertebrate and vertebrate models have been conducted to determine whether CUR was also neuroprotective [58]. A compelling new body of literature is also mounting to support the efficacy of CUR in stress and mood disorders. Current understanding of the biological basis for antidepressant-relevant biochemical and behavioral changes shows convergence with some mechanisms known for standard antidepressants [59].

Recently, Xiang et al. reported that THC regulates the oxidative stress response and aging via the O-type forkhead domain transcription factor (FOXO). In NIH3T3 cells, THC induced nuclear accumulation of FOXO4, a member of the FOXO family of transcription factors, by inhibiting phosphorylation of protein kinase B (PKB)/Akt. FOXO factors act as sensors in the insulin/IGF-1 (IIS) pathway and influence mammalian longevity. Overall, the totality of the evidence supports a potential role of FOXO3A in human health, aging, and longevity. The association of FOXO with diverse aging phenotypes, including insulin sensitivity, CHD, cancer, type 2 diabetes, and longevity, is suggestive of a “gatekeeper” role in the IIS pathway. An important downstream mechanism whereby FOXO3A might influence human aging is through modification of oxidative stress. In* D. melanogaster*, THC attenuated the oxidative stress response, an effect that was blocked in a FOXO mutant background. THC extended the life span of* Drosophila* under normal conditions, and loss of either FOXO or Sir2 activity eliminated this effect. Based on these results, it seems that THC may regulate the aging process via an evolutionarily conserved signaling pathway that includes both FOXO and Sir2 [60].

Pu et al. tested the hypothesis that dietary CUR, which has an antioxidant effect, can improve aging-related cerebrovascular dysfunction via mitochondrial uncoupling protein 2 UCP2 upregulation. Dietary CUR administration for one month remarkably restored the impaired cerebrovascular endothelium-dependent vasorelaxation in aging Sprague Dawley rats. In cerebral arteries from aging Sprague Dawley rats and cultured endothelial cells, CUR promoted eNOS and AMPK phosphorylation, upregulated UCP2, and reduced ROS production. These effects of CUR were abolished by either AMPK or UCP2 inhibition. Chronic dietary CUR significantly reduced ROS production and improved cerebrovascular endothelium-dependent relaxation in aging wild type mice but not in aging UCP2^−/−^ mice. CUR supplementation ameliorated age-associated large elastic artery stiffening, nitric oxide-mediated vascular endothelial dysfunction, oxidative stress, and increase in collagen and AGEs levels in mice [61].

Yanase et al. examined the effects of PAK1-deficiency or downregulation on a few selected functions of* C. elegans*, including reproduction, expression of HSP16.2 gene, and lifespan. They found that PAK1 promotes reproduction, whereas it inactivates HSP16.2 gene and shortens lifespan, as do PI-3 kinase (AGE-1), TOR, and insulin-like signalling/ILS (Daf-2) in this worm. These findings not only support the “trade-off” theory on reproduction versus lifespan, but also suggest the possibility that the reduced reproduction (or HSP16.2 gene activation) of this worm could be used as the first indicator of extended lifespan for a quick* in vivo* screening for PAK1-blockers [62]. Yu et al. examined the modulation of oxidative-stress resistance and associated regulatory mechanisms by CUR also in a* C. elegans* model. CUR-treated wild-type* C. elegans* exhibited increased survival during juglone-induced oxidative stress compared to the control treatment. In addition, CUR reduced the levels of intracellular ROS in* C. elegans*. CUR induced the expression of the gst-4 and hsp-16.2 stress response genes. Lastly, their findings from the mechanistic study in this investigation suggest that the antioxidant effect of CUR is mediated via regulation of age-1, akt-1, pdk-1, osr-1, unc-43, sek-1, skn-1, sir-2.1, and mev-1 [63].

In* D. melanogaster*, CUR, which extended the lifespan of* D. melanogaster*, also modulated the expression of several aging-related genes, including mth, thor, InR, and JNK [64]. Shen et al. found that lifespan extension by CUR in* Drosophila* was associated with the upregulation of Mn-SOD and CuZn-SOD genes and the downregulation of dInR, ATTD, Def, CecB, and DptB genes. These authors suggested that CUR increases mean lifespan of* Drosophila* via regulating gene expression of the key antioxidant enzyme SOD and reducing lipid peroxidation [65].

However, not always overexpression of antioxidant enzymes may be relevant for the lifespan. In particular, the overexpression of major antioxidant enzymes, which decrease the steady-state level of ROS, does not extend the lifespan of mice. Overexpression of SODs protects against oxidative stress but has little or no effect on the lifespan of* C. elegans *[66, 67]. The lifespan of sod-2 mutant of* C. elegans* was not decreased but even extended suggesting that ROS toxicity does not play a major role in lifespan regulation in these animals [68]. One possible explanation of why deletion of individual SOD genes failed to shorten lifespan is compensation by additional SOD genes. However, a recent report from the Hekimi lab demonstrates that worms lacking all five SOD genes are viable and have normal lifespan, despite significantly increased sensitivity to multiple stresses [69]. These observations indicate that oxidative damage caused by superoxide radical does not contribute to worm aging. It should be expected that species with weak antioxidant defense, accumulating oxidative damage, should be short lived, which is definitely not true for the longest living rodent, the naked mole rat* Heterocephalus glaber* [70].

The term “green tea” refers to the product manufactured from fresh tea leaves by steaming or drying at elevated temperatures with the precaution to avoid oxidation of the polyphenolic components known as catechins. The natural product EGCG accounts for 50–80% of catechins in green tea, representing 200–300 mg in a brewed cup of green tea. Several other catechins such as (−)-epicatechin-3-gallate (ECG), (−)-epigallocatechin (EGC), and (−)-epicatechin (EC) are found in lower abundance in green tea. EGCG is defined as a major green tea catechin that contributes to beneficial therapeutic effects, including antioxidant, anti-inflammatory, anticancer, and immunomodulatory effects [71].

EGCG binds strongly to many biological molecules and affects a variety of enzyme activities and signal transduction pathways at micromolar or nanomolar levels [72]. Most of the medicinal properties of green tea are associated with the “epicatechins” (2R, 3R) rather than the catechins (2S, 3R). The green tea catechins have been shown to be more effective antioxidants than Vitamins C and E, and their order of effectiveness as radical scavengers is ECG < EGCG < EGC < EC < catechin. The metal-chelating properties of green tea catechins are believed to be also important contributors to their antioxidative activity [73]. EGCG acts as a powerful hydrogen-donating radical scavenger of ROS and RNS and chelates divalent transition metal ions (Cu^2+^, Zn^2+^ and Fe^2+^), thereby preventing the Fe^2+^-induced formation of free radicals* in vitro*. Among 12 polyphenolic compounds, EGCG most potently inhibited Fe^2+^- mediated DNA damage and iron ascorbate-promoted lipid peroxidation of brain mitochondrial membranes. During ageing, total Fe^2+^ concentration increases in some brain regions that are involved in the pathogenesis of degenerative diseases, such as Alzheimer's, Parkinson's, and Huntington's disease. This Fe^2+^ accumulation obviously fosters the production of the highly reactive hydroxyl radicals (OH^*·*^), which attacks a large number of functional groups of the biomolecules in neurons. By chelating redox-active transition metal ions, the gallate groups of EGCG are thought to inhibit the Fenton-like-reaction mechanism [74]. Thus, the formation of OH^*·*^ is inhibited. Consequently, polyunsaturated fatty acids in, for example, mitochondrial membranes are protected from lipid peroxidation [75].

Results obtained by Weinreb et al. shed some light on the antioxidative-iron chelating activities of EGCG underlying its neuroprotective/neurorescue mechanism of action, further suggesting a potential neurodegenerative-modifying effect for EGCG. Their study sought a deeper elucidation of the molecular neurorescue activity of EGCG in a progressive neurotoxic model of long-term serum deprivation of human SH-SY5Y neuroblastoma cells. In this model, proteomic analysis revealed that EGCG (0.1–1 *μ*M) affected the expression levels of diverse proteins, including proteins related to cytoskeletal components, metabolism, and heat shock. EGCG induced the levels of cytoskeletal proteins, such as beta tubulin IV and tropomyosin 3, playing a role in facilitating cell assembly. Moreover, EGCG increased the levels of the binding protein 14-3-3 gamma, involved in cytoskeletal regulation and signal transduction pathways in neurons. EGCG decreased protein levels and mRNA expression of the beta subunit of the enzyme prolyl 4-hydroxylase, which belongs to a family of iron-oxygen sensors of hypoxia-inducible factor (HIF) prolyl hydroxylases that negatively regulate the stability and degradation of several proteins involved in cell survival and differentiation. Accordingly, EGCG decreased protein levels of two molecular chaperones that were associated with HIF regulation, the immunoglobulin-heavy-chain binding protein, and the heat shock protein 90 beta [76].* In vivo*, EGCG increased expression and activity of antioxidant enzymes, such as glutathione peroxidase, glutathione reductase, SOD, and CAT and inhibited prooxidative ones, such as monoamine oxidase (MAO)-B. The rat lifespan extension by EGCG was due to reduction of liver and kidney damage and improving age-associated inflammation and oxidative stress through the inhibition of transcription factor NF-*κ*B signaling by activating the longevity factors: forkhead box class O 3A (FOXO3A) and SIRT1 [77]. FOXO genes are the closest human homologues of* C. elegans* DAF-16. In* C*.* elegans*, DAF-16 increases the expression of manganese superoxide dismutase (SOD2), which converts superoxide to less damaging hydrogen peroxide and is a potent endogenous protector against free radicals, among other “antiaging” effects.* In vivo* studies show that oxidative lesions in DNA, proteins, and other tissues accumulated with age and feeding calorically restricted diets (a potent insulin sensitizer) to rodents and humans mitigate this damage [78]. Brown et al. showed that 25 *μ*M EGCG does not provoke a significant change in the intracellular ROS level of* daf-16* mutant* C. elegans*, while in the wild type strain ROS levels are significantly reduced by the flavonoid. This indicates that EGCG decreases ROS levels in the nematode in a DAF-16 dependent manner [79].

Meng et al. examined EGCG for its antiaging effect on human diploid fibroblasts. Fibroblasts treated with EGCG at 25 and 50 *μ*M for 24 h considerably increased CAT, SOD1, SOD2, and glutathione peroxidase gene expressions and their enzyme activities, thus protecting the cells against H_2_O_2_-induced oxidative damage, accompanied by decreased intracellular ROS accumulation and well-maintained mitochondrial potential. Moreover, fibroblasts treated with EGCG at 12.5 *μ*M for long term showed less intracellular ROS with higher mitochondrial potential, more intact mitochondrial DNA, much elevated antioxidant enzyme levels, and more juvenile cell status compared to those of the untreated group [80]. Davinelli et al. investigated the combined effect of L-carnosine and EGCG on the activation of two stress-responsive pathways: heme oxygenase (HO)-1 and Hsp72 (the inducible form of Hsp70), which play an important role in cytoprotection against oxidative stress-induced cell damage. They demonstrated that the neuroprotective effects of EGCG and L-carnosine are achieved through the modulation of HO-1/Hsp72 systems. Moreover, the combined action of both compounds resulted in a synergistic increase of HO-1 expression which suggests a crosstalk between the HO-1 and the Hsp72-mediated pathways [81]. Rodrigues et al. analyzed the neuroprotective effects of prolonged consumption of a green tea extract rich in catechins but poor in EGCG and other green tea bioactive components that could also afford benefit. Theses authors demonstrated that the consumption of an extract rich in catechins rather than EGCG protected the rat hippocampal formation from aging-related declines contributing to improving the redox status and preventing the structural damage observed in old animals, with repercussions on behavioral performance [82]. Feng et al. investigated the protective effects of EGCG on hydrogen peroxide (H_2_O_2_)-induced oxidative stress injury in human dermal fibroblasts. The incubation of human dermal fibroblasts with EGCG markedly inhibited the human dermal fibroblast injury induced by H_2_O_2_. The assay for 2,2-diphenyl-1-picrylhydrazyl radical scavenging activity indicated that EGCG had a direct, concentration-dependent antioxidant activity. Treatment of human dermal fibroblasts with EGCG significantly reversed the H_2_O_2_-induced decrease of SOD and glutathione peroxidase and the inhibition of malondialdehyde levels. These authors suggested that EGCG should have the potential to be used further in cosmetics and in the prevention of aging-related skin injuries [83].

In addition to the plethora of evidence that catechins are cytoprotective via antioxidant and antiapoptotic effects, recent observations suggest that the catechins may also contain prooxidant properties, particularly at high concentrations. Thus, at low concentrations* in vitro* (1–50 *μ*M), they are antioxidant and antiapoptotic, whereas at higher concentrations (100–500 *μ*M), the reverse is true. DNA isolated from humans was exposed to 200 *μ*M of EGC and EGCG, which induced oxidative damage due to the production of hydrogen peroxide. Green tea extract (10–200 *μ*g/mL) and EGCG (20–200 *μ*M) exacerbated oxidant activity, oxidative stress, genotoxicity, and cytotoxicity induced by hydrogen peroxide in RAW 264.7 macrophages [84]. Catechins, particularly EGCG (100 *μ*M), have also been shown to increase the oxidative damage incurred after exposure of DNA to 8-oxo-7,8-dihydro-2′-deoxyguanosine [85].

The lifespan-prolonging effect of catechin* in C. elegans* may be related to a significant reduction in body length and modulation of energy-intensive stress response [86]. The lifespan extension of* C. elegans* by apple procyanidins is dependent on SIR-2.1 as treatment with procyanidins had no effect on the longevity of SIR-2.1 worms, which lack the activity of SIR-2, a member of the sirtuin family of NAD+-dependent protein deacetylases [87].

Extension of lifespan of* D. melanogaster* by black tea extract seems to be at least partly due to increased expression of SOD and catalase (CAT) [88]. The analogous effect of black rice extract is most likely due to upregulating the genes of SOD1, SOD2, CAT, Mth, and Rpn11 at the transcriptional level [89]. The effects of flavonoids (myricetin, quercetin, kaempferol, and naringenin) on the lifespan of* C. elegans* involved an increased DAF-16 translocation and sod-3 promoter activity [90].

Longevity-promoting regimens, including DR and inhibition of TOR with rapamycin, RSV, or the natural polyamine spermidine, have often been associated with autophagy and in some cases were reported to require autophagy for their effects. Seemingly, clearing cellular damage by autophagy is a common denominator of many lifespan-extending manipulations [91].

Maintenance of optimal long-term health conditions is accomplished by a complex network of longevity assurance processes that are controlled by vitagenes, a group of genes involved in preserving cellular homeostasis during stressful conditions. Vitagenes encode for heat shock proteins (Hsp) Hsp32, Hsp70 the thioredoxin and the sirtuin protein systems. Dietary antioxidants, such as polyphenols, have been demonstrated to be protective through the activation of hormetic pathways, including vitagenes and proteasomal activity degrading oxidatively modified proteins [92, 93].

The life-prolonging effects of complex extracts are usually ascribed to the antioxidants present in these extracts but they may contain also toxins produced by plants against insects and microorganisms which may induce a hormetic effect [36]. Such a hormetic mechanism of action has been reported for the effects of* Ginkgo biloba* extract EGb 761 on the lifespan of* C. elegans *[94]. But perhaps antioxidants can also act via hormetic mechanisms and can belong to hormesis-inducing compounds (hormetins) [93]. Like toxins, they act in some concentration range, their high concentrations being usually toxic. A hormetic action of quercetin and other flavonoids on* C. elegans *has been documented [95]. It is debatable whether hormesis, which undoubtedly increases longevity of invertebrates, can be of relevance as an aging-delaying factor in mammals and especially in human but there are reasons to assume that it modulates “public” mechanisms of aging and delay aging of mammals even if these effects are not of a large magnitude [36].

Paradoxically, the effect of hormesis may be mediated by increased formation of ROS, especially by the mitochondria believed to be the main source of ROS in the cell. In the concept of mitochondrial hormesis (*mitohormesis*), increased formation of ROS within the mitochondria evokes an adaptive response that culminates in subsequently increased stress resistance assumed to ultimately cause a long-term reduction of oxidative stress. Mitohormesis was claimed to provide a common mechanistic denominator for the physiological effects of physical exercise, reduced calorie uptake, and glucose restriction [96]. This idea questions the FRTA and rather suggests that ROS act as essential signaling molecules to promote metabolic health and longevity [97].

The glycolytic inhibitor lonidamine (5 *μ*M) was found to extend both median and maximum lifespan of C.* elegans* by 8% each. This compound promotes mitochondrial respiration and increases formation of (ROS). Extension of lifespan is abolished by coapplication of an antioxidant, indicating that increased ROS formation is required for the extension of lifespan by lonidamine [98]. The same effects were found in* C. elegans* for low concentrations of arsenite [99], a cytotoxic and antimalarial quassinoid glaucarubinone [100], and glucose restriction [101].

In summary, complex effects of exogenous antioxidants in model organisms are compatible with the current understanding of the role of ROS, which are not only damaging agents but also take part in the signaling pathways and may mediate beneficial response reactions on the basis of hormetic mechanisms [102–104]. The direct antioxidant action of antioxidant supplements seems thereby to be much less important than induction of endogenous antioxidants, especially via the Nrf-2 dependent pathway [105].

## 5. Antioxidant Supplementation in Humans: Does It Make Sense?

The changes in the structure of contemporary human populations are characterized by an increase in the fraction of people who are 65 years and older, a phenomenon of significant importance from demographic, political, social, and health points of view [106]. Nutrition has been recognized to have an important impact on overall mortality and morbidity; and its role in extending life expectancy has been the object of extensive scientific research. Dietary supplementation with antioxidants has become more and more popular. However, their biochemical mechanisms of protection against oxidative stress and antiaging effects are not fully understood. The Mediterranean diet (MeDi), a heart-healthy eating plan that emphasizes fruits, vegetables, whole grains, beans, nuts, seeds, healthy fats, and red wine consumption rich in antioxidants like RSV which have been shown to have protective effects against oxidative damage [107]. The Mediterranean lifestyle has been for many millennia a daily habit for people in Western civilizations living around the Mediterranean sea who worked intensively and survived with very few seasonal foods. A high adherence to the traditional MeDi is associated with low mortality (higher longevity) and reduced risk of developing chronic diseases, including cancer, the metabolic syndrome, depression, and cardiovascular and neurodegenerative diseases [108]. Recently, several foodstuffs have been claimed as “antiaging”, principally on the basis of their anti-inflammatory and antioxidative properties: berries; dark chocolate; beans (due to their high concentration in low-fat protein, protease inhibitors, fibrins, genistein, and minerals); fish; vegetables; nuts; whole grains; garlic (due to the high amount of garlic-derived polysulfides that undergo catabolism to hydrogen sulfide promoting vasodilatation); and avocados (as a great source of monounsaturated fat, vitamins, and antioxidants) [109]. These authors reviewed the pathophysiological mechanisms that potentially link aging with diet and the scientific evidence supporting the antiaging effect of the traditional MeDi, as well as of some specific foods. Recently, five places [Okinawa (Japan), Sardinia (Italy), Loma Linda (California), Ikaria (Greece), and Nicoya (Costa Rica)] have been recognized as having a very high prevalence of octogenarians and have joined the Blue-Zones, a National Geographic project. Among the lifestyle habits that are common to those populations are high levels of daily physical activity (e.g., gardening and walking), positive attitude (e.g., an ability to articulate a sense of purpose and enriching their day with periods of calm and midday siesta), and a wise diet-high consumption of fruit, wild plants and vegetable, and low consumption of meat products. That diet is similar to the MeDi [110]. MeDi may not only reduce the risk for Alzheimer's disease [111], but also lower mortality rates and speed of disease progression in those already afflicted [112]. On the other hand, in a prospective cohort study of 1410 older adults, a higher adherence to MeDi did not lower the risk for incident dementia [113]. In another study, a higher adherence to MeDi failed to delay the transition from a cognitively healthy status to mild cognitive impairment [114]. Titova et al. suggested that one possible reason for these contrasting findings could be that the MeDi score, which is commonly used to explore correlations between MeDi and health outcomes in elderly cohorts, may mask health-related effects of certain dietary components by including others that are not relevant for the health domain of interest [115].

Most recently, Bacalini et al. discussed the potential impact of so-called “epigenetic diet” on age-related diseases, focusing on cardiovascular disease, highlighting the involvement of epigenetic modifications rather than DNA methylation, such as microRNA [116]. Epigenetic modifications may delay the aging process and impact diverse health benefits by activating numerous intracellular pathways. One leading theory suggests that bioactive phytochemicals including 1-isothiocyanato-4-(methylsulfinyl) butane (sulforaphane), (2R, 3R)-5,7-dihydroxy-2-(3,4,5-trihydroxyphenyl)-3-4-dihydro-2H-chromen-3-yl, 3,4,5-trihydroxybenzoate (epigallocatechin gallate), RSV, and CUR play significant roles as epigenetic modifiers [117, 118].

In recent years, the wealth of basic science research supporting RSV's potential to treat, delay, and even prevent age-related chronic diseases has led to a number of human clinical trials. As research in nonclinical populations becomes more common, disparity in dosing protocols and clinical endpoints will likely continue to cause conflicting findings. The range of daily RSV dosage used in clinical trials for healthy individuals (75 to 5000 mg) [110] would be expected to result in different clinical responses [119, 120]. Brown et al. confirmed this, demonstrating 2500 mg to be more effective than both lower (500 mg and 1000 mg) and higher dosages (5000 mg) in reducing plasma insulin-like growth factor-1 (IGF-1) concentrations [119]. Though 1000 mg RSV did not alter IGF-1 concentrations, it was sufficient to reduce insulin-like growth factor binding protein-3 (IGFBP-3) concentrations. This demonstrates that there may not be a single optimal dose of RSV, but rather the ideal dose may vary depending on the target outcome measures, which is not uncommon for various drugs. Further research is warranted to increase our understanding of the physiological responses of RSV before widespread use in humans can be promoted. Furthermore, chronic studies are an absolute must, as it is still unclear if RSV supplementation on the longer term is beneficial for overall health status [111]. A synthetic analogue of RSV, HS-1793, may be a new potent chemopreventive agent against human prostate and breast cancer cells [121, 122]. HS-1793 showed more potent anticancer effects in several aspects compared to RSV in MCF-7 (wild-type p53) and MDA-MB-231 (mutant p53) cells [122]. Moreover, HS-1793 may inhibit human prostate cancer progression and angiogenesis by inhibiting the expression of hypoxic condition induced HIF-1*α* protein and vascular endothelial growth factor (VEGF). HS-1793 showed also more potent effects than RSV on the cytotoxic effects on PC-3 cells [120, 122].

*Gnetum gnemon* is an arboreal dioecious plant that is cultivated in Indonesia. The seeds of this species mainly contain dimeric stilbenoid compounds [gnetin C, gnemonoside A, and gnemonoside D along with trans-RSV] the active form of RSV. Recent data show showed that the ethanolic extract of* G. gnemon* seeds inhibits endothelial senescence, suggesting that trans-RSV plays a critical role in the prevention of endothelial senescence [123]. Fleenor et al. suggested that gnetin may be a novel therapy for treating arterial aging in humans [124].

It should be noted that status elderly people are a very heterogeneous group. The nutrition situation of “young” seniors does generally not differ from the situation of working-age adults while institutionalized elderly people and those in need of care often show signs of a global malnutrition. The critical nutrients in the nutrition of the elderly particularly include vitamins B12 and D. Six percent of all elderly have a manifest and 10 to 30% a functional vitamin B12 deficiency. The main cause is vitamin B12 malabsorption resulting from a type B atrophic gastritis. The functional vitamin B12 deficiency and the associated hyperhomocysteinemia are risk factors for neurodegenerative diseases and accelerate bone loss. With increasing age, the vitamin D status is deteriorating. About 50% of the elderly living in private households is deficient in vitamin D; in geriatrics vitamin D, deficiency is more the rule than an exception. This is caused by a reduced endogenous biosynthesis, low UVB exposure, and a diet low in vitamin D. A vitamin D deficiency increases the risk for falls and fractures as well as the risk for neurodegenerative diseases. Also the overall mortality is increased [125].

On the other hand, up till now no prospective clinical intervention studies have been able to show a positive association between antioxidant supplementation and increased survival. More studies are needed to understand the interactions among single nutrient modifications (e.g., protein/amino acid, fatty acids, vitamins, phytochemicals, and minerals), the degree of DR, and the frequency of food consumption in modulating antiaging metabolic and molecular pathways and in the prevention of age-associated diseases. Meta-analysis of mortality data from 57 trials with a supplementation period of at least one year was published between 1988 and 2009, with sample sizes ranging from 28 to 39.876 (median = 423), yielding 246.371 subjects, and 29.295 all-cause deaths indicating that supplementation with vitamin E has no effect on all-cause mortality at doses up to 5.500 IU/d [79]. The last meta-analysis of randomized controlled human trials, and studies performed with rodents also do not support the idea that the consumption of dietary supplements can increase the lifespan of initially healthy individuals [91].

Most recently, Macpherson et al. reported that multivitamin-multimineral treatment has no effect on mortality risk [126]. Bjelakovic et al. noted that antioxidant supplements do not possess preventive effects and may be harmful with unwanted consequences to our health, especially in well-nourished populations. The optimal source of antioxidants seems to come from our diet, not from antioxidant supplements in pills or tablets. Even more, beta-carotene, vitamin A, and vitamin E may increase mortality. Some recent large observational studies now support these findings [127].

In summary, while beneficial effects of antioxidant supplements seem undoubtful in cases of antioxidant deficiencies, additional studies are warranted in order to design adapted prescriptions in antioxidant vitamins and minerals for healthy persons.

## Figures and Tables

**Table 1:** Effect of supplementation with natural and synthetic antioxidants on the lifespan of model organisms.

Organism	Additive	Parameter studied	Effect reported	Reference
*Saccharomyces cerevisiae, *budding yeast	Ascorbic acid	Replicative lifespan of SOD-1 deficient mutant	Partial restoration of normal replicative lifespan	[128]

*Saccharomyces cerevisiae *	Erythroascorbic acid	Replicative lifespan of wild-type yeast	Little effect	[24]

*Saccharomyces cerevisiae *	*α*-tocopherol, CoQ alone, or with *α*-tocopherol	Replicative lifespan	Decrease increased oxidative stress	[24]

*Paramecium tetraurelia *	Vitamin E	Clonal lifespan	Increase maximal (382 versus 256 fissions) at 1000 mg/L medium	[129]

*Paramecium tetraurelia *	Melatonin	Clonal lifespan		[130]

*Asplanchna brightwellii,*rotifer	Vitamin E (25 ug/mL)	Lifespan	Increase limited to the prereproductive stage [15]	[131]

*Philodina acuticornis odiosa*, rotifer	Indolepropionamide	Lifespan	Increase up to 3-fold	[13]

*Caenorhabditis elegans, *nematode	CoQ Vitamin E	Lifespan	Prolongation	[132]

*Caenorhabditis elegans *	200 *µ*g/mL vitamin E from hatching to day 3	Survival	Increase (17–23%)	[133]

*Caenorhabditis elegans *	*γ*-Tocopherol	Lifespan	Slight extension, no effect of *α*-tocopherol	[134]

*Caenorhabditis elegans *	*γ*-, or *α*-tocopherol	Lifespan	No effect	[134]

*Caenorhabditis elegans *	Polydatin, resveratrol-3-O-*β*-mono-D-glucoside	Mean lifespan	Increase by up to 31% and 62% under normal and acute stress conditions, respectively	[56]

*Caenorhabditis elegans *	Curcumin	Lifespan	Increase in *mev*-1 and *daf-*16 mutants	[135]

*Caenorhabditis elegans *	Quercetin, isorhamnetin, and tamarixetin	Lifespan	Increase by 11–16%	[136]

*Caenorhabditis elegans *	Quercetin-3-O-glucoside	Lifespan	Increase by low concentrations, decrease by high concentrations	[137]

*Caenorhabditis elegans *	Myricetin, quercetin, kaempferol, and naringenin	Lifespan	Increase	[90]

*Caenorhabditis elegans *	Caffeic acid, and rosmarinic acid	Lifespan	Increase	[138]

*Caenorhabditis elegans *	Catechin	Mean lifespan, median lifespan	Increase by 9 and 13%, respectively, at 200 *μ*M	[86]

*Caenorhabditis elegans *	(−)-Epicatechin	Lifespan	No effect	[87]

*Caenorhabditis elegans *	Epigallocatechin gallate (220 nM)	Mean lifespan	Increase by 10%	[139]

*Caenorhabditis elegans *	Epigallocatechin gallate	Mean lifespan	Increase under stress conditions but not under normal conditions	[140]

*Caenorhabditis elegans *	Ferulsinaic acid (0.5–100 *μ*M)	Lifespan	Increase	[141]

*Caenorhabditis elegans *	Procyanidins from apples (*Malus pumila,*65 *µ*g/mL)	Mean lifespan	Increase	[87]

*Caenorhabditis elegans *	Tyrosol	Lifespan	Increase	[21]

*Caenorhabditis elegans *	Mn-N,N′-bis(salicylidene) ethylenediamide chloride (EUK-8), an SOD mimetic	Lifespan	Extension but only after specific culture conditions	[142]

*Caenorhabditis elegans *	KPG-7, a herb complex	Lifespan	Prolongation	[143]

*Caenorhabditis elegans *	EGb 761, extract of *Ginkgo biloba* leaves	Mean lifespan	Prolongation	[94]

*Caenorhabditis elegans *	Royal gelly	Lifespan	Prolongation	[34]

*Caenorhabditis elegans *	Pt nanoparticles (a SOD/CAT mimetic)	Lifespan	Prolongation	[31]

*Drosophila melanogaster *	Lipoic acid	Lifespan	Increase	[144]

*Drosophila melanogaster *	Melatonin	Lifespan	Increase	[145]

*Drosophila melanogaster *	Epitalon	Lifespan	Increase by 11–16%	[14]

*Drosophila melanogaster *	Carnosine	Average lifespan	Increase in males, no effect on females	[15]

*Drosophila melanogaster *	*S,S*-Trolox-carnosine	Average lifespan	Increase in males (by 16%) and in females (by 36%)	[15]

*Drosophila melanogaster *	Curcumin, 1 mg/g of medium	Lifespan	Increase	[146]

*Drosophila melanogaster *	Curcumin, 0.5 and 1.0 mg/g	Lifespan	Increase by 6% and 26% in females and by 16% and 13% in males	[65]

*Drosophila melanogaster *	Curcumin	Lifespan	Extension, gender- and genotype-specific	[64]

*Drosophila melanogaster *	*Aloe vera* extract	Lifespan	Extension	[147]

*Drosophila melanogaster *	Extract of black rice	Lifespan	Increase by ca 14%	[89]

*Drosophila melanogaster *	Cacao	Lifespan	Increase	[88]

*Drosophila melanogaster *	Black tea extract	Mean lifespan	Increase by 10%	[148]

*Drosophila melanogaster *	EUK-8 Mn 3-methoxy-N,N′-bis(salicyldene) ethylenediamine chloride (EUK-134), mitoquinone	Lifespan	No effect on wild type flies	[149]

*Anastrepha ludens, *Mexican fruit fly	*γ*-, or *α*-Tocopherol	Lifespan	No effect	[134]

*Mus musculus*, mouse, strain C57BL/6	Vitamin E, lifelong	Median lifespan	Increase by 15%	[150]

*Mus musculus *	Vitamin E	Lifespan	No effect	[151]

*Mus musculus*, C3H/He and LAF1	Vitamin E, 0.25% w/w	Lifespan	Increase in mean lifespan, no effect on maximum lifespan	[152]

*Mus musculus*, SAMP8 (senescence-acceleration prone)	Lipoic acid	Lifespan	Decrease	[45]

*Mus musculus*, SAMP8	Melatonin	Lifespan	Increase	[153]

*Mus musculus *	Tetrahydrocurcumin, 0.2% from the age of 13 m	Average lifespan	Increase	[20]

*Mus musculus*, males from the age of 12 m	LGcombo, complex mixture of botanical extracts, vitamins, and nutraceuticals	Lifespan	No effect	[33]

*Mus musculus *	A fullerene mimetic of SOD	Lifespan	Prolongation	[32]

*Mus musculus *	Royal gelly	Lifespan	Increased mean lifespan, no effect on maximal lifespan	[35]

*Rattus rattus, *rat, Wistar	Epigallocatechin gallate	Median lifespan	Increase by 8–12 weeks (control: 105 weeks)	[77]

*Microtus agrestis*, field vole	Vitamin C or vitamin E, for 9 m	Lifespan	Decrease	[28]

*Mus musculus, Ellobius talpinus* (mole vole), *Phodopus campbelli *(dwarf hamster)	SkQ1 (mitochondrially targeted plastoquinone derivative)	Survival	Increase	[23]
